# Neurotrophic Factor-α1/Carboxypeptidase E Functions in Neuroprotection and Alleviates Depression

**DOI:** 10.3389/fnmol.2022.918852

**Published:** 2022-05-26

**Authors:** Lan Xiao, Yoke Peng Loh

**Affiliations:** Section on Cellular Neurobiology, Eunice Kennedy Shriver National Institute of Child Health and Human Development, National Institutes of Health, Bethesda, MD, United States

**Keywords:** neurotrophic factor-α1, carboxypeptidase E, neuroprotection, depression, neurodegeneration

## Abstract

Depression is a major psychiatric disease affecting all ages and is often co-morbid with neurodegeneration in the elderly. Depression and neurodegeneration are associated with decreased neurotrophic factors. In this mini-review the functions and potential therapeutic use of a newly discovered trophic factor, Neurotrophic factor-α1 (NF-α1), also known as Carboxypeptidase E (CPE), in depression and neuroprotection are discussed. NF-α1/CPE expression is enriched in CA3 neurons of the hippocampus. Families carrying null and homozygous non-sense mutations of the NF-α1/CPE gene share common clinical features including childhood onset obesity, type 2 diabetes, impaired intellectual abilities and hypogonadotrophic hypogonadism. Studies in animal models such as CPE knockout (KO) mice and CPE*^fat/fat^* mutant mice exhibit similar phenotypes. Analysis of CPE-KO mouse brain revealed that hippocampal CA3 was completely degenerated after weaning stress, along with deficits in hippocampal long-term potentiation. Carbamazepine effectively blocked weaning stress-induced hippocampal CA3 degeneration, suggesting the stress induced epileptic-like neuronal firing led to the degeneration. Analysis of possible mechanisms underlying NF-α1/CPE -mediated neuroprotection revealed that it interacts with the serotonin receptor, 5-HTR1E, and *via* β arrestin activation, subsequently upregulates ERK1/2 signaling and pro-survival protein, BCL2, levels. Furthermore, the NF-α1/CPE promoter contains a peroxisome proliferator-activated receptor (PPARγ) binding site which can be activated by rosiglitazone, a PPARγ agonist, to up-regulate expression of NF-α1/CPE and neurogenesis, resulting in anti-depression in animal models. Rosiglitazone, an anti-diabetic drug administered to diabetic patients resulted in decline of depression. Thus, NF-α1/CPE is a potential therapeutic agent or drug target for treating depression and neurodegenerative disorders.

## Introduction

Depression is one of the most devastating and prevalent neuropsychiatric diseases that affect millions of people globally (Fava and Kendler, 2000). Clinical symptoms include anhedonia, feelings of sadness, loss of interest in life, sleep problems, idea of suicide, and impaired cognitive function (Nestler et al., 2002). Unfortunately, the pathophysiology of depression is still not fully understood. Indeed, scientists have found that hippocampus and several other brain regions that are critical for regulating mood, sleep and eating are altered at molecular and cellular levels in depression. Dysfunction of hypothalamic-pituitary-adrenal axis, glucocorticoids, and neurotrophic factors have been reported to be closely associated with the development of major depression (Nestler et al., 2002; Neto et al., 2011). Emerging studies have suggested that neurotrophic factors play an important role in protecting neurons against stress-induced cell death and promoting survival. This is supported by studies from both clinical patients with depression and animal models: in patients with major depression disorders, the volume of limbic system such as hippocampus was significantly smaller compared with control (Sheline et al., 1996; Neumeister et al., 2005; Campbell and MacQueen, 2006; Drevets et al., 2008; MacQueen et al., 2008). In addition, significant loss of neurons and glia and increased apoptosis were found in post-mortem brain of patients with depression (Lucassen et al., 2001; Krishnan and Nestler, 2008). All these changes in depression are accompanied with a decrease in several neurotrophic factors such as brain derived neurotrophic factor (BDNF) (Duman and Monteggia, 2006; Bocchio-Chiavetto et al., 2010; Yang et al., 2020), nerve growth factor (NGF) (Ueyama et al., 1997; Chen et al., 2015), glial derived neurotrophic factor (GDNF) (Lin and Tseng, 2015; Zhang et al., 2018; Sun et al., 2019), carboxypeptidase E (CPE)/neurotrophic factor-α1 (NF-α1) (Cheng et al., 2015), vascular endothelial growth factor (VEGF) (Elfving et al., 2010), non-acronymic (VGF) (Thakker-Varia et al., 2007; Cattaneo et al., 2010) and fibroblast growth factor 2 (FGF2) (Evans et al., 2004; Gaughran et al., 2006). Effective treatment with antidepressants increased the levels of some of these neurotrophic factors (Mallei et al., 2002; Hunsberger et al., 2007; Thakker-Varia et al., 2007; Warner-Schmidt and Duman, 2007; Sen et al., 2008; Cattaneo et al., 2010; Cheng et al., 2015) in depression, suggesting they are involved in the pathophysiology of depression and could be good targets or biomarkers for depression.

Among these neurotrophic factors, NF-α1/CPE is a newly identified neurotrophic factor, and studies on its trophic function are still limited, but expanding. NF-α1/CPE was initially found to be an exopeptidase that processes proneuropeptides and prohormones by cleaving the C-terminal basic amino acids from endoproteolytically cleaved intermediates (Hook et al., 1982; Fricker, 1988). Intrigued by the high concentration of CPE, equivalent to peptide hormone levels and much higher than other prohormone processing endoproteases in the secretory vesicles of (neuro)endocrine cells, we proposed that secreted CPE may have an extracellular trophic role. Early *in vitro* studies demonstrated secreted CPE’s ability to protect rat primary hippocampal neurons from oxidative stress-induced cell death, providing the first evidence that it has a neurotrophic role (Cheng et al., 2013). Hence it was given an alternative and more appropriate name, NF-α1. In this mini-review, studies on the function of NF-α1/CPE in neuroprotection and depression, from preclinical animal models to clinical patients, its receptor and downstream signaling cascade and mechanism in mediating neuroprotection and anti-depression effects are discussed.

## Role of Neurotrophic Factor-α1/Carboxypeptidase E and its Receptor in Neuroprotection

In 1992, the CPE gene was identified to be located on chromosome 4q32.3 in human (Hall et al., 1993). Patients with null and homozygous non-sense mutations of the CPE gene have been identified, and they present with neuroendocrinological abnormalities such as childhood onset obesity, type 2 diabetes, hypogonadotropic hypogonadism, and intellectual disabilities (Alsters et al., 2015; Bosch et al., 2021; Durmaz et al., 2021). In transgenic mice with CPE gene knock-out (KO) or CPE*^fat/fat^* mice carrying a Ser202Pro mutation, similar phenotypes such as obesity, infertility and diabetes were also characterized (Rodriguiz et al., 1993; Naggert et al., 1995; Leiter, 1997; Cawley et al., 2004, 2012). In addition to the endocrinological abnormalities, impairments in cognitive function and depression-like behaviors were observed in transgenic mouse models with CPE mutations (Hall et al., 1993; Cheng et al., 2013) suggesting the critical role of CPE in neurodegenerative and neuropsychiatric disorders.

Studies in CPE-KO mice revealed abnormalities at several levels: Behaviorally, CPE-KO demonstrated learning disability and depression-like behaviors (Woronowicz et al., 2008). Electrophysiological analysis showed hippocampal long-term potentiation was compromised in CPE-KO mice (Woronowicz et al., 2008). Morphological analysis showed that the hippocampal CA3 region, where CPE is highly expressed, was degenerated after weaning stress which included maternal separation and physical stress, such as ear tagging and tail clipping for genotyping. In contrast, this CA3 region in the CPE-KO mice was completely intact at 3 weeks of age before weaning, indicating that weaning stress induced hippocampal CA3 degeneration (Woronowicz et al., 2008). Interestingly, this degeneration was prevented by oral administration of an anti-epileptic drug, carbamazepine, at 50 mg/kg daily starting at age of 2 week old for 2 weeks (Woronowicz et al., 2012); indicating that the weaning stress, which upregulates glucocorticoid secretion and induces epileptic-like neuronal firing of the granule cells in the dentate gyrus to increase glutamate secretion leading to excitotoxicity, likely caused the death of the hippocampal CA3 neurons in the CPE-KO mice (Woronowicz et al., 2012). Studies *in vitro* corroborated the hypothesis that CPE produced the neuroprotective effect by enhancing neuronal survival. Hippocampal neurons in culture from CPE-KO mice are more prone to die in comparison with neurons from WT control, and treatment with recombinant CPE significantly reversed the high death rate (Cheng et al., 2013). Additionally, pretreatment with recombinant CPE or overexpression of CPE protected hippocampal or cortical neurons from hydrogen peroxide- or glutamate- induced toxicity by activating ERK1/2 (extracellular-signal-regulated kinase) and AKT signaling cascades, upregulating pro-survival mitochondrial protein, Bcl2, and inhibiting caspase 3 activation (Cheng et al., 2013). Furthermore, rosiglitazone, a ligand for peroxisome proliferator-activated receptors (PPARγ), has been reported to exert neuroprotective effects (Thouennon et al., 2015), and enhanced hippocampal neurogenesis by upregulating CPE expression *via* binding to the CPE promoter (Cheng et al., 2015).

**FIGURE 1:**
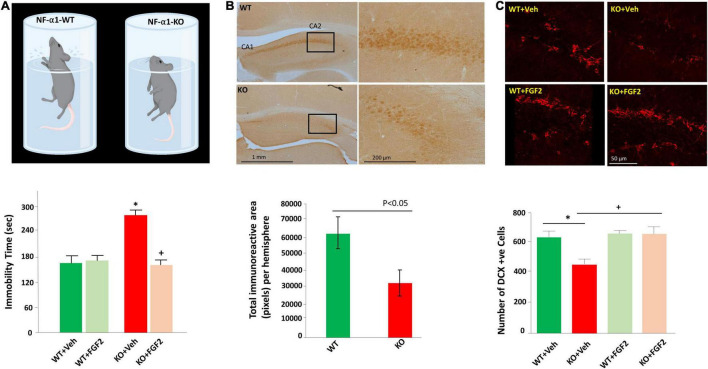
FGF2 reversed impaired hippocampal neurogenesis and depression-like behaviors in NF-α1/CPE-KO mice. **(A)** Diagram (upper panel) illustrates the depressive-like behavior of the NF-α1-KO mice versus WT mice in the forced swim test. Bar graph (lower panel) shows immobility time of the forced swim test was significantly increased in NF-α1-KO mice, compared with WT controls, indicating depressive-like behavior. FGF2 treatment reversed immobility time to normal level in NF-α1-KO mice. **(B)** Immunocytochemistry (upper panel) and bar graph (lower panel) show FGF2 protein expression was decreased in the hippocampal CA1 and CA2 regions of NF-α1-KO in contrast to WT control. **(C)** Immunofluorescence imaging (upper panel) and bar graph (lower panel) show doublecortin (DCX) positive immature neurons in the subgranular zone (SGZ) of the dentate gyrus was decreased in NF-α1-KO mice, in contrast to WT control, however, FGF2 treatment, but not vehicle, rescued impaired neurogenesis in NF-α1-KO mice. *p < 0.05 KO+Veh compared with WT+Veh. ^+^*p* < 0.05 KO+FGF2 compared with WT+FGF2. Figure reproduced from Cheng et al. (2015), with permission from Springer Nature.

Further examination of the mechanism underlying CPE-mediated neuroprotection indicated that the glucocorticoid agonist, dexamethasone, remarkably increased expression of CPE protein and mRNA in rat hippocampal neurons (Murthy et al., 2013). Bioinformatic and luciferase studies revealed that a glucocorticoid receptor-binding site existed in the -1460 to -1442 region of CPE promoter, and thus NF-α1/CPE expression can be upregulated by high glucocorticoid level induced by stress (Murthy et al., 2013). Studies *in vivo* confirmed that mild chronic restraint stress for 1 h daily for 7 days significantly elevated levels of NF-α1/CPE in the hippocampus, particularly in CA3 area. Evaluation of downstream pathway showed that in addition to increased NF-α1/CPE, mild chronic stress also increased Bcl2, decreased Bax, and enhanced phosphorylation of AKT in WT mice. However, in CPE-KO mice, mild chronic stress decreased Bcl2, increased Bax and reduced phosphorylated AKT in the hippocampus (Murthy et al., 2013). The inverse changes in Bcl2/AKT/Bax between WT and CPE-KO mice suggest an important role of CPE/NF-α1 in neuroprotection during stress. Besides, FGF2 has been also reported to be involved in the CPE-mediated neuroprotective activity. FGF2 was profoundly decreased in the hippocampus of CPE-KO mice, which displayed reduced neurogenesis and CA3 degeneration (Figure 1B). Interestingly, treatment with recombinant CPE significantly increased FGF2 at both mRNA and protein levels in primary hippocampal neurons (Cheng et al., 2015). Taken together, NF-α1/CPE is a key neurotrophic factor for protecting hippocampal CA3 neurons against stress-induced death.

Binding analysis showed that CPE/NF-α1 labeled with ^125^I bound specifically to cell membranes of an immortalized mouse hippocampal cell line HT22 in a saturable manner with a Kd 4.37 nM. This indicated involvement of a receptor-mediated mechanism in CPE’s action. Further studies revealed that inhibitors of tropomyosin receptor kinase B (TrkB) and fibroblast growth factor receptor (FGFR1-3), K235a and PD16285, respectively, had no effect on NF-α1/CPE -mediated neuroprotective activity in H_2_O_2_-treated hippocampal neurons, suggesting a disparate type of receptor is required (Xiao et al., 2021). Recent studies from high throughput screening of a human G protein coupled receptors (GPCR) library revealed a promising candidate, 5-hydroxytryptamine receptor 1E (5-HTR1E), as a potential binding partner for NF-α1/CPE. Interestingly, this receptor is expressed in humans and non-human primates and guinea-pig (Leonhardt et al., 1989; Bruinvels et al., 1994; Bai et al., 2004; Klein and Teitler, 2009), but not in mice and rats. Molecular modeling studies predicted that NF-α1/CPE interacts with 5-HTR1E, outside of its serotonin pocket, and stabilized by several salt bridges and hydrogen bonds. Moreover, molecular dynamic studies revealed strong coupling of the intracellular loop 3 of HTR1E with β-arrestin1, indicating potential activation of β-arrestin1 (Sharma et al., 2021). Immunohistochemical analysis of human hippocampus showed that NFα1/CPE and 5-HTR1E were colocalized on the cell membrane of neurons, suggesting a functional role. In addition, co-immunoprecipitation, and pull-down assays in HEK293 cells indicate that NFα1/CPE binds with 5-HTR1E in a saturable, high-affinity manner, with a Kd 13.82 nM. Cell biological studies demonstrated that NF-α1/CPE exerted protective activity against oxidative stress in HEK293 cells by interacting with 5-HTR1E and activating downstream β-arrestin/ERK/CREB/BCL2 signaling cascade (Sharma et al., 2021). Furthermore, studies showed that NFα1/CPE treatment decreased cytotoxicity induced by oxidative stress in primary human neurons, but not when HTR1E expression was knocked-out in these neurons (Sharma et al., 2021). These *ex vivo* studies indicated the physiological role of HTR1E-NFα1/CPE interaction in neuroprotection of human neurons. Currently, the function of NFα1/CPE and 5-HTR1E interaction *in vivo* in the central nervous system remains unexplored. As well, the role of HTR1E in the physiological and pathological changes in neurodegenerative and neuropsychiatric disorders such as depression is not known. Therefore, more future studies are needed to shed light on the function of the NFα1/CPE-5-HTR1E interaction *in vivo*.

## Role of Neurotrophic Factor-α1/Carboxypeptidase E in Anti-Depressant Activity

Emerging studies have indicated that NF-α1/CPE plays a significant function in the development of depression. Restraint stress, which has been used widely in research on depression, is an effective and valid model of inducing depression-like behaviors (Chu et al., 2016; Hwang et al., 2020). For instance, long term chronic restraint stress for 6 h daily for 21 days induced depression-like behaviors in mice as evidenced by increased immobility in forced-swim test (Cheng et al., 2015). Simultaneously, biochemical analysis showed decreased levels of NFα1/CPE, 21 Kd and 24 Kd FGF2, as well as decreased hippocampal neurogenesis in mice challenged with long term chronic restraint stress. Interestingly, in CPE-KO mice lacking NFα1/CPE also showed decreased 17 Kd and 24 Kd FGF2 levels and reduced hippocampal neurogenesis, in addition to depression-like behavior in forced swim test (Figures 1A,C) and sucrose preference test. Subcutaneous injection of FGF2 at a dose of 5 ng/g for 30 days significantly reversed depression-like behaviors and impaired hippocampal neurogenesis in CPE-KO mice (Cheng et al., 2015). *In vitro*, NFα1/CPE treatment demonstrated enhanced FGF2 expression at both mRNA and protein levels in rat hippocampal neurons. However, this effect was blocked by ERK inhibitor U0126, transcription inhibitor actinomycin D and Sp1 inhibitor, mithramycin A, but not AKT inhibitor LY294002, suggesting that NFα1/CPE exerts its effect on up-regulating FGF2 expression *via* ERK-Sp1 signaling cascade (Cheng et al., 2015).

Furthermore, studies showed that rosiglitazone and pioglitazone which are peroxisome proliferator-activated receptor gamma (PPARγ) agonists produced antidepressant-like effects in both animal model and clinical patients. In mice exposed to unpredictable chronic mild stress, rosiglitazone significantly reversed depressive-like behaviors in forced swim test and open field test (Zhao et al., 2017). In addition, rosiglitazone conferred antidepressant-like effects in animals, as evidenced by decreased immobility in rat forced swim test and mouse tail suspension test (Eissa Ahmed et al., 2009). A clinical study in 12 patients with depressive disorder and insulin resistance showed that add-on treatment with rosiglitazone for 12 weeks effectively relieved the depressive symptoms and decreased depression score (Rasgon et al., 2010). It also has been reported that pioglitazone at 30 mg per day for 12 weeks and 24 weeks mitigated depressive symptoms in clinical patients (Kemp et al., 2009; Roohafza et al., 2014). Recent analysis including 4 open label studies and 4 randomized control trials with 448 patients diagnosed with major depression suggested that pioglitazone and rosiglitazone alone or add-on for 6 to 12 weeks significantly alleviated depressive symptoms, as well as improved three glucose intolerance biomarkers (Colle et al., 2017). Regarding the underlying mechanism of these drugs, rosiglitazone has been shown to activate PPARγ which then binds to the PPARγ regulatory elements (PPREs) in the CPE promoter to activate transcription of NFα1/CPE mRNA in neurons (Thouennon et al., 2015). Animal studies revealed that rosiglitazone exerted its anti-depressant effect by upregulating hippocampal CPE expression and neurogenesis, suggesting a possible role of NFα1/CPE in the treatment of depression (Cheng et al., 2015).

Moreover, a mutation of the CPE gene located in expressed sequence tag (EST) has been identified in the cortex of AD patients (Kimura et al., 2006). This mutation introduced three adenosine inserts into the CPE gene, and thus replaced eight amino acids with nine new amino acids. The mutated CPE contained two adjacent glutamine residues, and thus named as CPE-QQ. Transgenic mice carrying CPE-QQ mutation demonstrated depression-like behaviors and typical pathological changes associated with neurodegeneration such as memory deficits, reduced dendrites in hippocampus and prefrontal cortex, increased hyperphosphorylation of the microtubule-associated protein tau at ser395 and decreased hippocampal neurogenesis. Further studies in mouse neuroblastoma cell line Neuro2a revealed that overexpression of CPE-QQ resulted in accumulation of the mutant protein in the cell and was not secreted. When WT-CPE was co-expressed with CPE-QQ in Neuro2A cells, secretion of WT-CPE was reduced by 78.4%. Remarkably, ER stress marker, CHOP was much higher in cells overexpressing CPE-QQ, indicating CPE-QQ might accumulate abnormally in the ER and lead to ER stress and neurotoxicity (Cheng et al., 2016). Collectively, these studies showed that introduction of this mutated human CPE gene into a transgenic mouse model gave rise to neurodegeneration, impairment of memory, as well as depressive-like behavior, indicating the pivotal role of NFα1/CPE in neurodegenerative diseases and depression.

## Conclusion and Future Research

**FIGURE 2:**
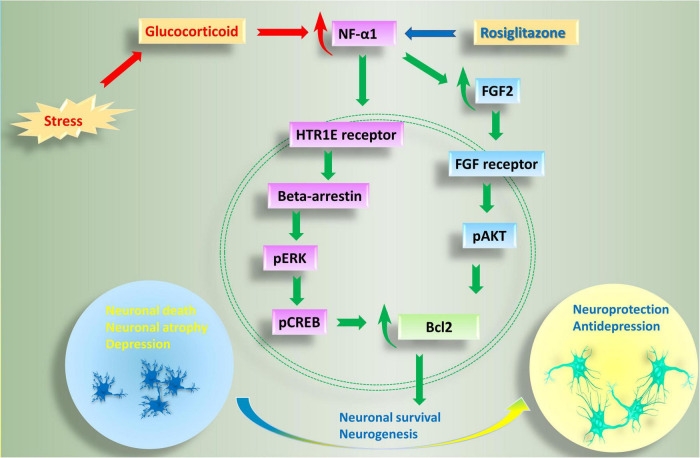
Anti-depressive and neuroprotective effects of NF-α1 in the central nervous system. Long term chronic stress induces depression-like behaviors, as well as a reduction in NF-α1 and FGF2 levels in the hippocampus. Decreased NF-α1 and FGF2 signaling leads to impaired neuroprotection and increased neuronal death and associated neurodegenerative disease and depression. In contrast, short term chronic stress, as well as the drug, rosiglitazone, upregulate NF-α1 expression, enhance both NF-α1 and FGF2 signaling cascades, neuronal survival and neurogenesis. NF-α1 binds to a receptor such as HTR1E on the cell membrane which then activates beta-arrestin. Recruitment of beta-arrestin enhances pERK/pCREB and Bcl2, leading to increase in neuronal survival. On the other hand, NF-α1 can also regulate FGF2 expression in a positive manner. When FGF2 is increased, both pERK/pCREB and pATK signaling pathways, which converge on Bcl2, are upregulated and neuronal survival is enhanced; additionally, there is increase in neurogenesis leading to anti-depressant effects. HTR1E, hydroxytryptamine receptor 1E; FGF2, fibroblast growth factor 2; pCREB, phosphorylation of cAMP response element binding protein.

Studies thus far indicate that NFα1/CPE plays a key role in preventing stress-induced neurodegeneration and depression. The mechanism involves binding of NFα1/CPE to a receptor such as HTR1E, to activate ERK signaling pathway and increase in levels of BCL2, a mitochondrial pro-survival protein, to mediate neuroprotection; and enhancement of neurogenesis *via* up-regulation of FGF2 in the dentate gyrus (Figure 2). Preclinical and preliminary clinical studies indicate that NFα1/CPE is a potential therapeutic agent for treating neurodegenerative disorders such as Alzheimer’s disease (AD) and major depressive disorder (MDD). Indeed, similar to using neurotrophic factors such as NGF (Tuszynski and Gage, 1990; Tuszynski et al., 2015) and BDNF (Nagahara et al., 2018; Tuszynski, 2021) in gene therapy or infusion of the protein, future clinical trials with administering NFα1/CPE protein, or Adeno-associated virus carrying NFα1/CPE mRNA into the hippocampus could prove to be efficacious in treating these disorders.

Another treatment approach in treating AD and depression has been to use drugs that stimulate the production of neurotrophic factors. A class of drugs that has given some positive outcomes in treating such disorders in patients are the thiazolidinediones: rosiglitazone and pioglitazone which have traditionally been used to treat Type 2 diabetes (Rasgon et al., 2010; Kashani et al., 2013). These drugs are PPARγ agonists and have been shown to increase NFα1/CPE expression which then have anti-depressant effects. While long term use of these drugs have been shown to have side effects on cardiac function, with rosiglitazone more so than pioglitazone, short term use in multiple clinical studies suggest that these thiazolidinediones may be useful in treating depression and AD through enhancing neurotrophic factor expression (Rasgon et al., 2010; Kashani et al., 2013; Saunders et al., 2021). Future development of new classes of safe drugs that can enhance neurotrophic factor expression could be an excellent approach to treat depression and neurodegenerative diseases. Other antidepressants such as serotonin or noradrenaline re-uptake inhibitors, or monoamine oxidase inhibitors, have also been shown to elevate neurotrophin levels (Duman and Voleti, 2012). In summary, evidence reviewed herein support the importance of the new neurotrophic factor, NFα1/CPE, in ameliorating depression and neurodegeneration; hence future treatment strategies should explore stimulating NFα1/CPE expression, either by drugs or gene therapy, as well as finding agonists for the HTR1E, a receptor for NFα1/CPE.

## Author Contributions

LX wrote the manuscript. YL revised the manuscript. Both authors contributed to the article and approved the submitted version.

## Conflict of Interest

The authors declare that the research was conducted in the absence of any commercial or financial relationships that could be construed as a potential conflict of interest.

## Publisher’s Note

All claims expressed in this article are solely those of the authors and do not necessarily represent those of their affiliated organizations, or those of the publisher, the editors and the reviewers. Any product that may be evaluated in this article, or claim that may be made by its manufacturer, is not guaranteed or endorsed by the publisher.
